# A Comprehensive Review of Congenital Platelet Disorders, Thrombocytopenias and Thrombocytopathies

**DOI:** 10.7759/cureus.11275

**Published:** 2020-10-31

**Authors:** Gisha Mohan, Srikrishna V Malayala, Parth Mehta, Mamtha Balla

**Affiliations:** 1 Medical Research, Physicians for American Healthcare Access, Philadelphia, USA; 2 Internal Medicine, Temple University Hospital, Philadelphia, USA; 3 Internal Medicine, Unity Point Health Methodist Hospital, Peoria, USA; 4 Internal Medicine, ProMedica Toledo Hospital, Toledo, USA

**Keywords:** inherited platelet disorder, inherited diseases, thrombocytopenia, congenital abnormalities

## Abstract

Platelets play an important role in hemostasis through platelet plug formation by a phenomenon of adhesion; activation; secretion and aggregation. Defects in platelet hemostatic mechanisms can be congenital or acquired. Congenital platelet disorders are rare and manifestations range from asymptomatic to sometimes severe bleeding. The disorders arise due to diverse mechanisms. Congenital platelet disorders include thrombocytopathies and thrombocytopenia (platelet count <150 x 10^9^/L) or thrombocytosis (platelet count > 450 x 10^9^/L). Congenital thrombocytopathies include disorders of adhesion like von Willebrand's disease or Bernard-Soulier syndrome. The disorders of aggregation include congenital afibrinogenemia and Glanzmann thrombasthenia. Disorders of storage granules are gray platelet syndrome and Quebec platelet disorder. Congenital thrombocythopathy and thrombocytopenia often occur in conjunction. In this article, we have a detailed literature review of these rare thrombocytopathies, their presentation and treatment.

## Introduction and background

Platelets are an essential factor in primary hemostasis through platelet plug formation, leading to secondary hemostasis by forming a coagulation cascade. von Willebrand's disease is the most common bleeding disorder and has been well studied and reported in the medical literature. The other rare Congenital thrombocytopathias include Bernard-Soulier syndrome, Glanzmann thrombasthenia, Gray platelet syndrome, Chediak-Higashi syndrome, and Scott syndrome. This review article provides a detailed analysis of the rarest thrombocytopathias, including Gray platelet syndrome, Glanzmann thrombasthenia, and Bernard-Soulier syndrome.

## Review

Platelet plug formation begins with an injury leading to endothelial damage and follows a sequence of adhesion, activation, aggregation, and secretion leading to a balanced action of pro-aggregation and anti-aggregation factors [1-3]. von Willebrand factor (vWF) is present inside endothelial cells. Once there is an injury, vWF is released from the vascular endothelial cells and alpha granules of platelets, and it binds with subendothelial collagen. At the specific site of injury, glycoprotein Ib (GpIb) platelets bind with vWF, which results in a conformational change of platelets, resulting in the release of adenosine diphosphate (ADP), thromboxane A2, and calcium ions. ADP has two roles. First, it helps with the adhesion of platelets to the endothelium. Second, it binds to the P2Y12 receptor, resulting in glycoprotein IIb/IIIa (GPIIb/IIIa) expression at the platelet surface. GPIIb/IIIa on platelet surfaces are the key for platelet aggregation and are bound with fibrinogen's help [4,5]. 

**Figure 1:**
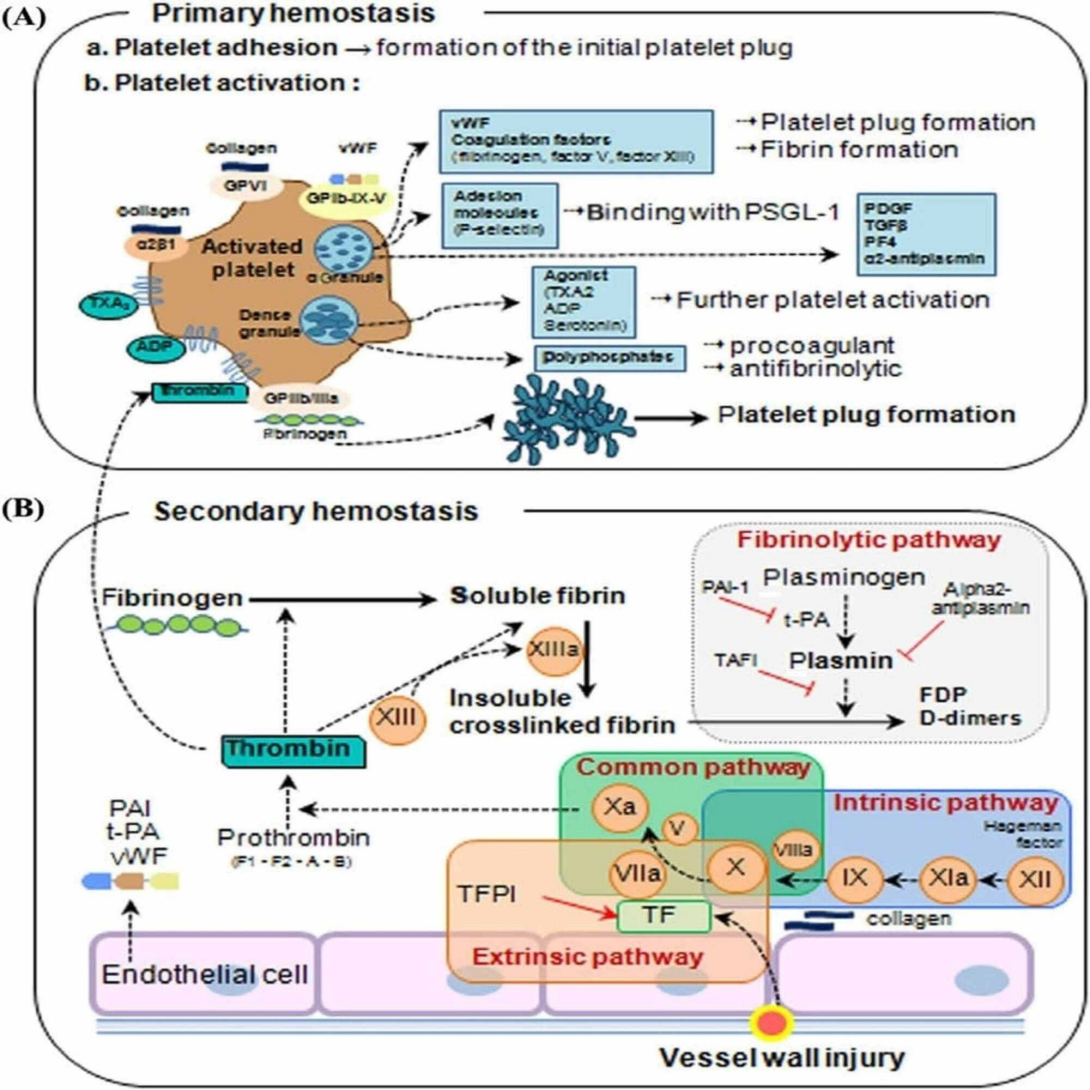
Overview of hemostasis (A) Primary hemostasis: the formation of the primary platelet plug. When the endothelium is injured, the procoagulant subendothelial matrix (consisting of proteins, such as collagen, von Willebrand factor (vWF), fibrinogen (FBG), laminin, and fibronectin) is exposed, and the subendothelial matrix proteins bind to glycoprotein (GP) receptors on the platelet surface and immediately initiate primary hemostasis, consisting of (1) platelet adhesion, (2) platelet activation, and (3) platelet plug formation. vWF binding to the GPIb-IX-V complex, collagen binding to platelet GPVI, and integrin receptors trigger a signal transduction process resulting in the local release of platelet activation agonists from dense granules, such as thromboxane A2 (TXA2) and ADP. These agonists, along with thrombin produced from coagulation cascades and activated platelets, bind to platelet surface-bound G-coupled receptors, inducing further platelet activation. Dense granules also contain polyphosphates, which are both procoagulant (i.e., they promote the cofactor activity of factor V in the common pathway) and anti-fibrinolytic (i.e., they help to form dense fibrin fibrils that are more resistant to fibrinolysis). Activated platelets release vWF and coagulation factors from granules, which lead to platelet plug formation (vWF and fibrinogen) and fibrin formation (factor V and factor XIII). This helps platelets bind firmly to the endothelium and allows leukocytes to be incorporated into developing clots. The granule content also includes platelet-derived growth factor (PDGF), transforming growth factor (TGF-), and platelet factor 4 (PF4). Activation of platelet integrin IIb 3 induces platelet aggregation mediated by fibrinogen/vWF. (B) Secondary hemostasis (formation of fibrin by coagulation proteins). In the extrinsic pathway, vessel wall injury leads to the expression of tissue factor (TF) on endothelial cells. TF complexes with factor VIIa to activate factors X and Xa [5].

This primary cascade results in the activation of the secondary coagulation cascade (Figure 1).

**Figure 2:**
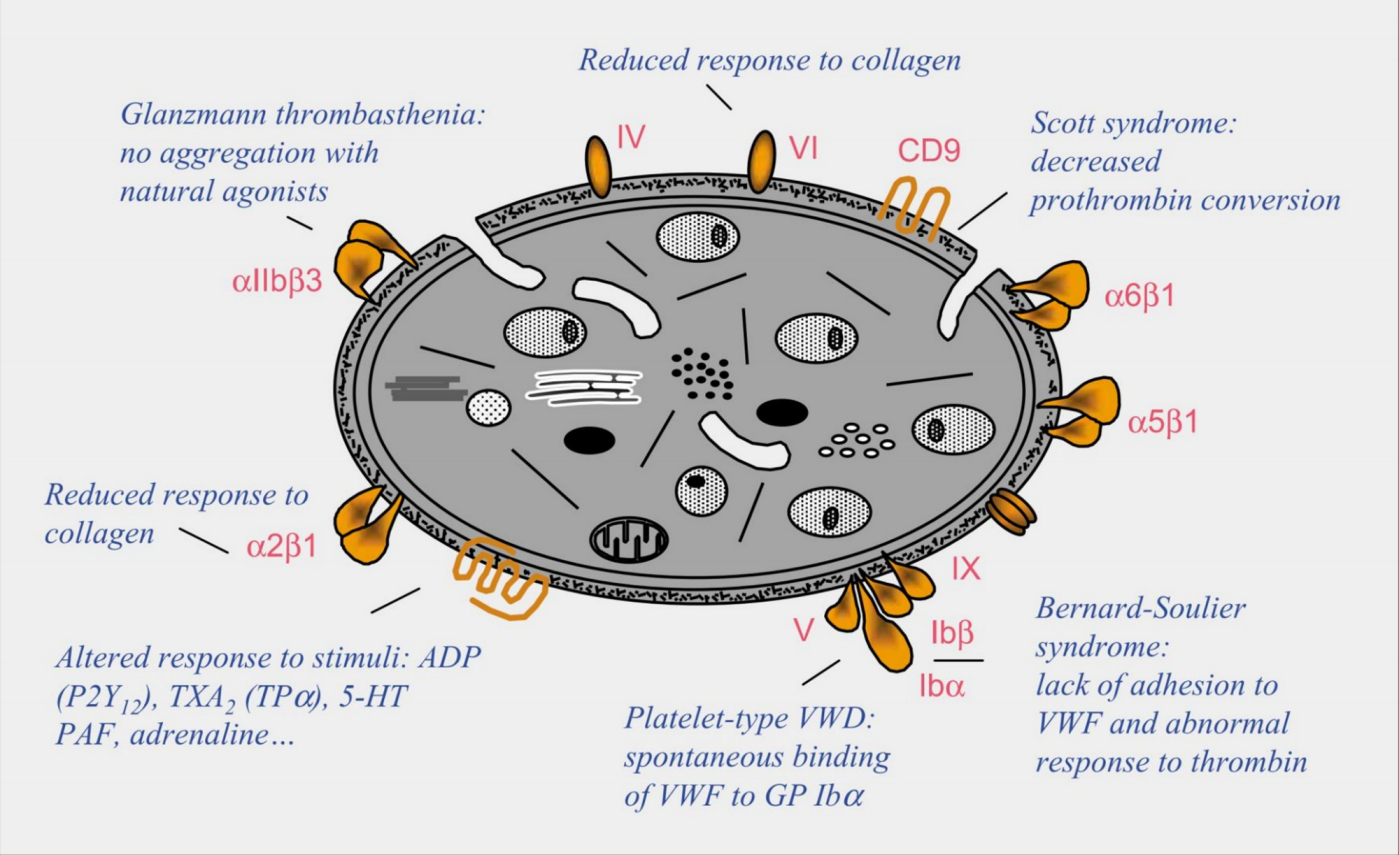
Disorders that mainly affect surface components of platelets

**Figure 3:**
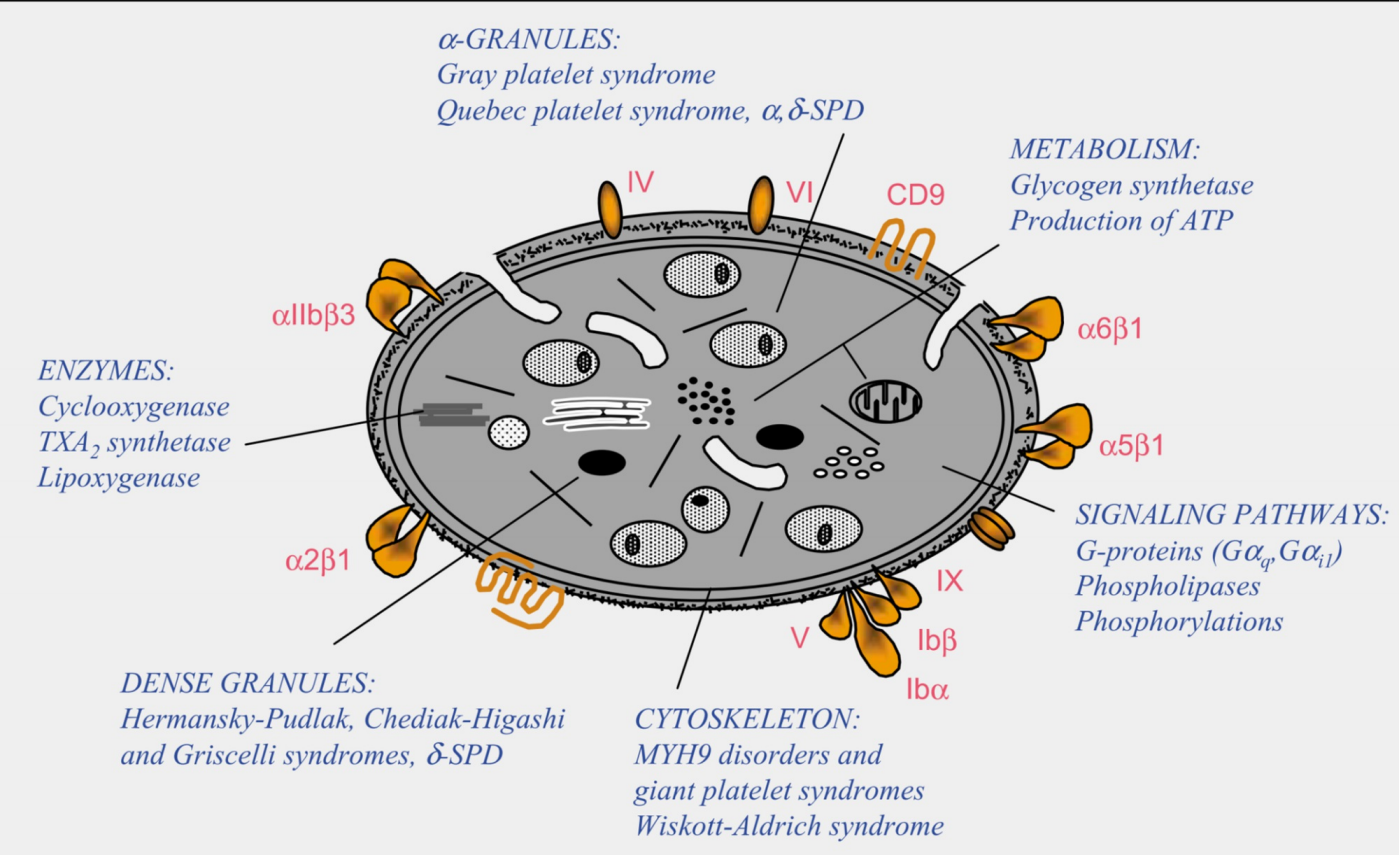
Disorders that mainly affect intracellular components of platelets

Platelet disorders can be congenital or acquired, although congenital platelet disorders are very rare. Platelet disorders are classified as disorders in platelet function, also known as thrombocytopathia and disorders in platelet numbers, i.e. thrombocytopenia [6]. There are platelet disorders that mainly affect surface components of platelets and disorders that mainly affect intracellular components of platelets (Figures 2, 3).

Bernard-Soulier syndrome

Bernard-Soulier syndrome was named after Dr. Jean Bernard and Dr. Jean Soulier in 1948, for their discovery of a rare autosomal recessive (AR) congenital thrombocytopathy in a young patient who presented with prolonged bleeding time, thrombocytopenia, and large platelets [1]. It is an extremely rare disease occurring in less than one person per one million population [7]. Bernard-Soulier syndrome is also referred to as giant platelet disorder or Hemorrhagiparous thrombocytic dystrophy characterized by large platelets. The condition is more frequent in ethnic groups, such as the Iraqi population and in regions where consanguineous marriages are common such as in the south of India and northern Iran [8].

Symptoms

Bernard-Soulier syndrome often presents similar symptoms to bleeding disorders characterized by abnormal menstrual bleeding, epistaxis, ecchymosis, and mucosal bleeding. Homozygous patients may have more severe symptoms and uncontrolled bleeding following trauma. Heterozygous carriers are often asymptomatic.

Genetics

Bernard-Soulier syndrome is a platelet adhesion disorder that stems from the qualitative or quantitative defects of the glycoprotein IbIX/V (GPIbIX/V) complex, which is present on the platelet surface and serves as a receptor for vWF binding, resulting in platelet adhesion and agglutination, leading to primary clot formation. The GPIbIX/V complex's biosynthesis and functioning require GPIbα, GPIbβ, and GPIX [1,7].

Although most Bernard-Soulier syndrome cases are identified to have autosomal recessive inheritance, there are two cases identified with GPIBA mutation transmitted via autosomal dominant trait that has been described [8]. Patients with classical Bernard-Soulier syndrome are homozygous or compound heterozygous for mutations in the GPIbα, GPIbβ, or GPIX genes. Patients in the heterozygous group are often asymptomatic. Forty-seven different genetic defects associated with Bernard-Soulier syndrome have been identified. The first type of defect identified is the point mutation, the most common type, which results in premature stop signals and unstable glycoprotein complex with decreased surface expression. The second type of defect identified is a mis-sense mutation of residue 57, leading to a valine to alanine substitution at position 1567, affecting the surface domain [9-11].

Laboratory Diagnosis

**Figure 4:**
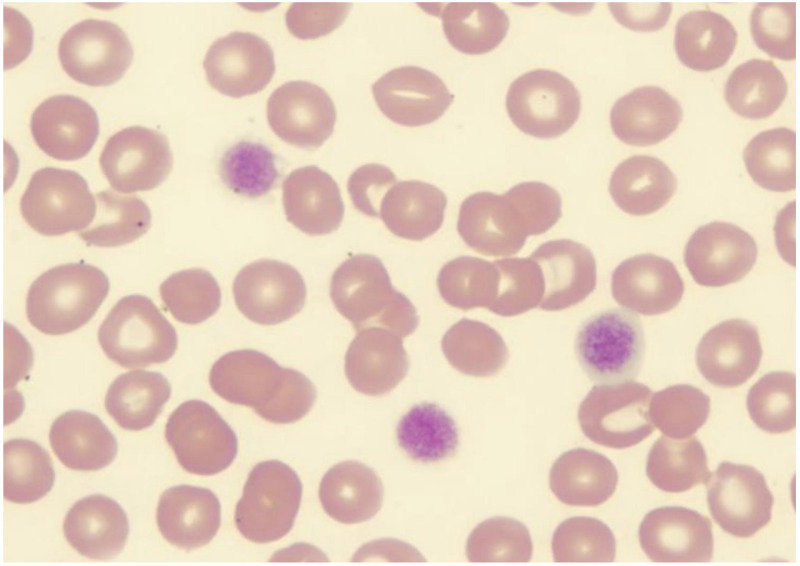
Giant platelets in Bernard Soulier syndrome Giant platelets of Bernard Soulier syndrome in the peripheral smear

Complete blood count reveals thrombocytopenia with a platelet count ranging from 10,000 to 280,000/microliter in homozygous patients and 20,000 to normal values in heterozygous patients [12]. Peripheral blood smear reveals large platelets with a rounded shape (Figure 4).

The failed agglutination of platelets to ristocetin, which is not corrected by the addition of plasma, is the distinctive feature and differentiates it from other platelet disorders, especially von Willebrand disease [13]. Flow cytometry confirms the diagnosis, and further gene defects can be diagnosed with the help of molecular studies.

Treatment

For minor bleeding, antifibrinolytic agents such as tranexamic acid can be used. For major surgeries, platelet transfusion is sometimes necessary. Human leukocyte antigen-matched, leukocyte-depleted platelets are used for transfusion to prevent transfusion-related antibody-mediated reactions. Caution should be used while using nonsteroidal anti-inflammatory drugs (NSAIDs), as aspirin can inhibit platelet aggregation and further worsen the condition. Genetic counseling is necessary for family and patients with a history of Bernard-Soulier syndrome. New studies are ongoing to assess the efficacy of recombinant-activated factor VIII and hematopoietic stem cell transplantation in Bernard-Soulier syndrome treatment [11-13].

Glanzmann thrombasthenia

Glanzmann thrombasthenia is a rare AR disease affecting the GPIIb/IIIa receptor and is associated with a defect in platelet aggregation. The condition was first discovered by Swiss pediatrician Eduard Glanzmann and was subsequently credited for this [14]. He also discovered the familial pattern and hereditary component of the disease [14]. The incidence/prevalence of Glanzmann thrombasthenia is approximately one per one million population worldwide and is more prevalent in the Middle East, including individuals of Palestinian descent and those in Israel, Iran, Iraq, Saudi Arabia, India, Jordan, and France [15,16]. Glanzmann thrombasthenia is more prevalent in women than in men, and the average age at diagnosis is eight years [17]. This disease, with defects in platelet aggregation, affects the megakaryocyte lineage.

Clinical Features

Clinical features include mucosal bleeding such as epistaxis, gingival bleeding, purpura, abnormal menstrual bleeding, and subcutaneous hematomas. A few cases manifest bleeding symptoms early after birth, although most patients are diagnosed later in life [14]. Studies have shown that even though severe bleeding can occur at any age, the risk and prevalence increases with age [15]. Symptoms are most prominent in homozygous or compound heterozygous patients. Heterozygous phenotypes with 50% to 70% functional αIIbβ3 show no significant reduction in platelet aggregation and are mostly asymptomatic [5,18].

Molecular Genetics

Glanzmann thrombasthenia is characterized by quantitative and qualitative defects in GPIIb/IIIa (ITG αIIbβ3, aka αIIbβ3 integrin). The cases are seen more often in consanguineous marriages. The αIIbβ3 integrin GPIIb/IIIa, CD41/CD61 is a large heterodimeric calcium-dependent molecule and is present in the platelet fibrinogen receptor, subsequently mediates platelet aggregation or thrombus at sites of endothelial injury. Henceforth, the defect in the same results in defective platelet aggregation and diminished clot retraction [14-16]. Glanzmann thrombasthenia is caused by mutations in either the GP2B (ITGA2B) or GP3A (ITGB3) gene, and at least 100 mutations have been described and collected in an online database [17-19].

Laboratory Diagnosis

The complete blood count shows normal platelet count and sometimes may show iron deficiency anemia. The prothrombin time and activated partial thromboplastin time will also be normal, but the bleeding time will be prolonged. To assess the platelet function and aggregation, light transmission aggregometry is being used and is a very specific test for the same. In Glanzmann thrombasthenia, platelet aggregation fails to occur with any agonist, except ristocetin, where the reaction is preserved. The platelet function analyzer (PFA) is the sensitive test for detecting Glanzmann thrombasthenia and is prolonged in Glanzmann thrombasthenia patients [20,21]. Flow cytometry shows decreased levels of CD41 and CD61 but normal levels of CD42. Mutation analysis is often helpful in diagnosing a defective mutation in Glanzmann thrombasthenia and is confirmed by a second DNA sample analysis [22].

Treatment

Glanzmann thrombasthenia patients require symptomatic treatment. All patients should be counseled about the cautious use of NSAIDs and aspirin and to avoid contact sports. Treatment during surgical procedures and controlling bleeding after injury and during spontaneous bleeding episodes is required. Blood transfusions may be necessary for severe bleeding situations; therefore, immunization against hepatitis B is advised in patients. Focused treatment after a bleeding episode entails the use of local measures such as the use of compression, fibrin sealants, and topical thrombin, alone or in conjunction with antifibrinolytic therapy, including tranexamic acid and epsilon-aminocaproic acid first, followed by platelet transfusion, and recombinant factor IIa if bleeding persists. Patients with severe bleeding episodes should continue to receive platelet transfusions for 48 hours after the cessation of bleeding, and until wound healing has occurred in patients undergoing surgery. The PFA assay time will normalize with adequate transfusions [19,22,23].

Gray platelet syndrome

Introduction

Gray platelet syndrome is an inherited disease of megakaryocytic lineage (i.e., alpha storage pool disease). The disease was initially described in 1971, and it is characterized by moderate thrombocytopenia, large abnormal platelets, and epistaxis. Gray platelet syndrome is rare, and only approximately 60 cases have been described [24-26]. 

**Figure 5:**
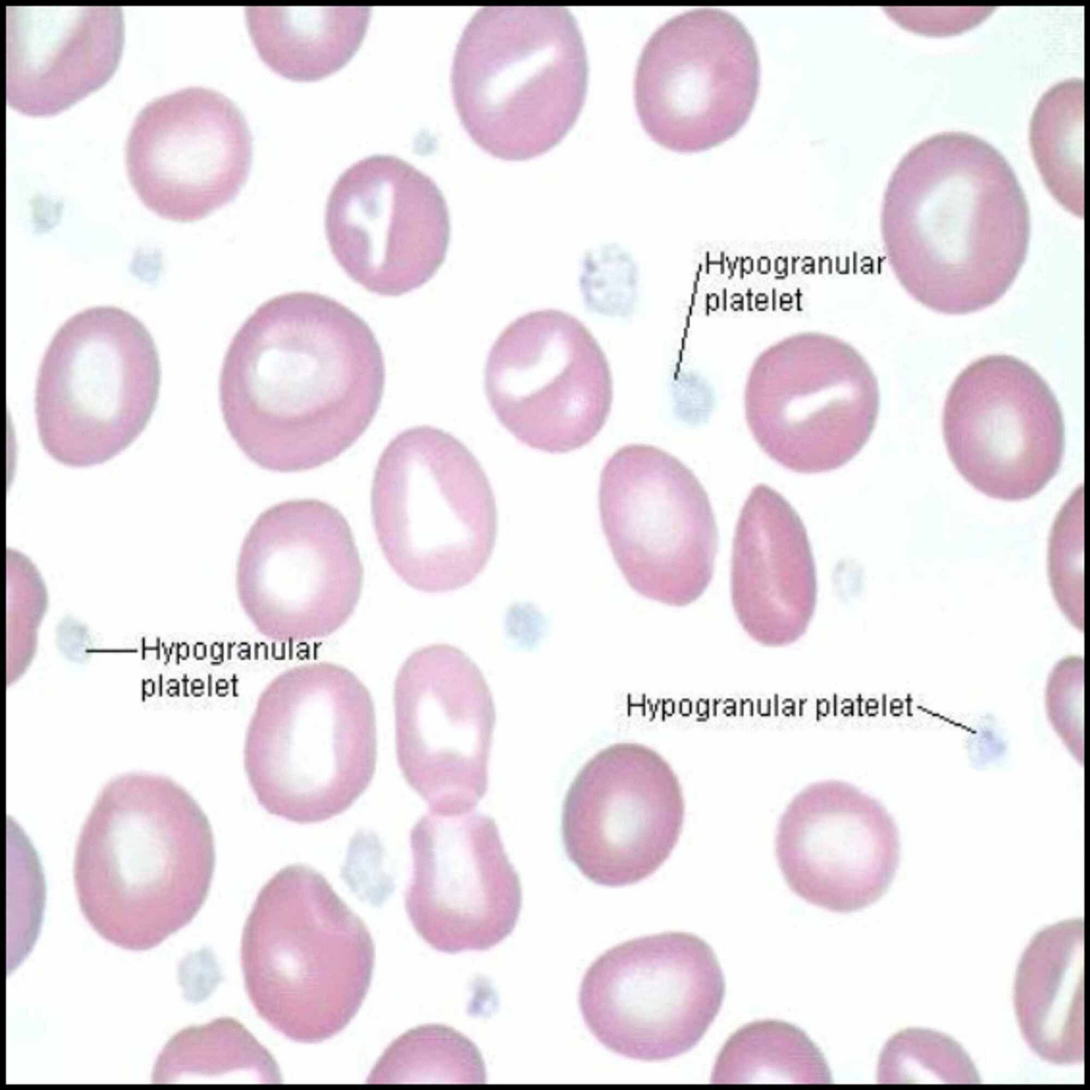
Hypogranular platelets in Gray platelet syndrome

Platelets contain dense granules and alpha granules (Figure 5).

The dense granules have ADP and calcium. The alpha granules consist of vWF, fibrinogen, and fibronectin. Alpha granules in platelets store proteins that help in platelet adhesion and wound healing once the platelet is activated after cell injury [25,26] and is found to be due to the inefficiency of the megakaryocyte to pack secretory proteins into alpha granules. Alpha granules, the most abundant vesicles in platelets, store proteins that promote platelet adhesiveness and wound healing when secreted during platelet activation [25,26]. The basic defect in Gray platelet syndrome is thought to be the inability of megakaryocytes to pack endogenously synthesized secretory proteins into developing α-granules.

Symptoms

The age of onset is usually at birth or early childhood. The severity of bleeding increases with increasing age, and symptoms include easy bruising, prolonged bleeding, epistaxis, and fatal hemorrhage in adulthood. Women usually have menorrhagia. Gray platelet syndrome is also associated with macrothrombocytopenia, myelofibrosis, and splenomegaly [26-28]. Myelofibrosis is due to the inability of defective megakaryocytes to mature, resulting in the absence of alpha granules in platelets [26,28].

Molecular Genetics

The inheritance pattern is found to be AR in the majority of patients with the neurobeachin-like-2 gene. X-linked inheritance is also described with the mutation of growth factor 1b. 

Laboratory Diagnosis

The complete blood count shows fewer platelets as well as increased vitamin B12 in some cases. A peripheral blood smear shows gray, large platelets in a Wright-stained peripheral blood smear, and an electron microscope shows absent or decreased alpha granules in platelets. 

Treatment

Treatment includes platelet transfusion before surgery and sometimes 1-desamino-8-D-arginine vasopressin. Splenectomy is suggested if the platelet count is less than 30,000/microliter. Genetic counseling is advised. In the case of an AR pattern, there is a chance of a 25% inheritance [25-28].

## Conclusions

The pathogenesis of congenital platelet disorders is complicated but has been better understood over the years. Many advances have been made in understanding the molecular characters of these disorders that have helped to have a very accurate understanding of congenital thrombocytopathias and thrombocytopenias. Accurate physical examination and appropriate laboratory tests are of great value for evaluating a patient presenting with bleeding due to congenital platelet disorders.
